# Towards an Understanding of *Mesocestoides vogae* Fatty Acid Binding Proteins’ Roles

**DOI:** 10.1371/journal.pone.0111204

**Published:** 2014-10-27

**Authors:** Gabriela Alvite, Natalia Garrido, Alejandra Kun, Margot Paulino, Adriana Esteves

**Affiliations:** 1 Sección Bioquímica, Facultad de Ciencias, Universidad de la República, Montevideo, Uruguay; 2 Instituto de Investigaciones Biológicas Clemente Estable, Montevideo, Uruguay; 3 Centro de Bioinformática Estructural-DETEMA, Facultad de Química, Universidad de la República, Montevideo, Uruguay; Simon Fraser University, Canada

## Abstract

Two fatty acid binding proteins, MvFABPa and MvFABPb were identified in the parasite *Mesocestoides vogae* (Platyhelmithes, Cestoda). Fatty acid binding proteins are small intracellular proteins whose members exhibit great diversity. Proteins of this family have been identified in many organisms, of which Platyhelminthes are among the most primitive. These proteins have particular relevance in flatworms since *de novo* synthesis of fatty acids is absent. Fatty acids should be captured from the media needing an efficient transport system to uptake and distribute these molecules. While HLBPs could be involved in the shuttle of fatty acids to the surrounding host tissues and convey them into the parasite, FABPs could be responsible for the intracellular trafficking. In an effort to understand the role of MvFABPs in fatty acid transport of *M. vogae* larvae, we analysed the intracellular localization of both MvFABPs and the co-localization with *in vivo* uptake of fatty acid analogue BODIPY FL C_16_. Immunohistochemical studies on larvae sections using specific antibodies, showed a diffuse cytoplasmic distribution of each protein with some expression in nuclei and mitochondria. MvFABPs distribution was confirmed by mass spectrometry identification from 2D-electrophoresis of larvae subcellular fractions. This work is the first report showing intracellular distribution of MvFABPs as well as the co-localization of these proteins with the BODIPY FL C_16_ incorporated from the media. Our results suggest that fatty acid binding proteins could target fatty acids to cellular compartments including nuclei. In this sense, *M. vogae* FABPs could participate in several cellular processes fulfilling most of the functions attributed to vertebrate’s counterparts.

## Introduction

Fatty acid binding proteins (FABPs) are small intracellular lipid binding proteins (iLBPs). FABP family members exhibit low similarity in terms of primary structure but have a highly conserved 3D structure, suggesting that they share a common ancestry [1]–[6].

They are characterised by high-affinity non-covalent binding of hydrophobic ligands, mainly fatty acids, having a mass of 14–15 kDa and folding in a characteristic β-barrel structure. A typical FABP consists of 127–134 amino acid residues with ten β strands folded into a barrel capped by two helices [7]. The ligand-binding site, e.g., for a fatty acid (FA), is within the large water-filled interior [8].

FABPs from vertebrates are named according to the tissue in which they were first identified or are predominantly expressed [9]. For example, those expressed in the liver belong to the subfamily named liver fatty acid binding proteins (L-FABPs), and those expressed in heart are named heart fatty acid binding proteins (H-FABPs). Different types within a species may share between 20–70% of sequence identity, whereas the members of the same FABP subfamily from different vertebrate species may share greater than 95% of identity [10].

The large diversity of FABPs has intrigued scientists for decades, and it is now clear that there are both unique and overlapping functions for specific FABPs [11]. Distinctive patterns of expression for vertebrate FABP subfamily members have been found, further supporting the idea that individual types are responsible for distinct functions in FA transport and metabolism [11]–[12]. Structural differences within the family and the inherent consequences for their binding interactions with fatty acids have been extensively studied [13]. Binding specificities vary from one subfamily member to another [14]. Important advances in the understanding of the function of individual FABPs have recently emerged [15]–[18]. However, the overall picture still remains incomplete.

More than 50 FABPs genes have been found in a wide range of invertebrates [19], but functional studies still remains in their infancy. Many features make parasitic platyhelminthes FABPs interesting molecules to study. These parasites are incapable of *de novo* synthesis of most of their own lipids, including long chain fatty acids and cholesterol, and in order to survive they depend largely on acquisition and utilisation of host’s FAs during infection to survive [20]. In this respect, FABPs could play an important role in facilitating the incorporation and intracellular distribution of host’s fatty acids.

Two highly similar FABP genes, *Mvfabpa* and *Mvfabpb*, have been identified in *Mesocestoides vogae* (syn. corti; Platyhelmithes, Cestoda). Curiously, both genes are expressed at the same larval stage suggesting that MvFABPa and MvFABPb may play distinct functions in the parasite and/or may be subject to differential regulation [21].

Though *Mesocestoides vogae* is not a public health threat, it is an important model organism because it shares similarities with taenia which are of public health interest. This parasite is easy to maintain in the laboratory by intraperitoneal passages through male mice and produces a very large number of larvae (tetrathyridia). The parasitic material obtained with this procedure is more homogenous, from a genetic point of view, than that derived from natural infections [22]. Likewise, the method of propagation in experimental animals allows the possibility of proteomic studies of particular genes, thus contributing to the elucidation of FABPs functions in these parasites.

Since subcellular localization could be indicative of putative functions, we studied the intracellular distribution of both MvFABPs at larval stage using two complementary approaches: a) confocal microscopy of larvae sections using specific antibodies and subcellular markers, b) MvFABPs mass spectrometry identification from subcellular fractions submitted to 2D electrophoresis. To investigate the role of these proteins in FA capture and intracellular targeting, we studied the co-localization of MvFABPa and MvFABPb with fluorescent fatty acid analogue BODIPY FL C_16_ incorporated *in vivo*. In addition, putative 3D nuclear localization signals were investigated.

The study of subcellular localizations of the two *M. vogae* proteins could help to elucidate the role of these proteins in lipid metabolism, gene expression regulation, and host-parasite relationships, as well as help to infer ancestral functions of this family of proteins.

## Materials and Methods

### Cloning strategy

*Mvfabpa* and *Mvfabpb* genes have been previously cloned, but 30 base pairs of the 5′ coding regions were missing in these constructs [21]. To obtain the complete coding sequences of both genes, inverse polymerase chain reaction was employed [23]. The following primers were designed: fw-MvFABPa 5′-GTTTTTGCATCACCTCGT C-3′; rev-MvFABPa 5′-GTGCAGATCGTTAAGGTAG-3′; fw-MvFABPb 5′-ACGCATCACCTCGTCGAAA-3′; rev-MvFABPb 5′GACAGTGAGTAGTGATTGC-3′. Primers were synthesised by SBS Genetech Co. Ltd. Beijing, China.

*M. vogae* DNA was extracted using a GFX DNA purification kit (GE Healthcare Life Sciences, Uppsala, Sweden), spectrophotometrically quantified and analysed using agarose gel electrophoresis. One µg of DNA was digested with Rsa I and Mbo I (Amersham Biosciences, Uppsala, Sweden), for 12 hours. The enzymes were inactivated at 65°C for 20 minutes. The quality of the digested DNA was analysed by electrophoresis. Digested fragments were then ligated using T4 DNA ligase (Fermentas, Vilnius, Lithuania) for 12 hours at 4°C. The mixture was precipitated with 3 M CH_3_COONa, pH 4.8 and 2 volumes of cold ethanol, and washed with 70% ethanol. DNA was solubilised in 65 µl of milliQ H_2_O and stored at −20°C until use.

PCR reactions were performed using a Perkin Elmer Gene Amp PCR System 2400. Reactions were performed in a total volume of 25 µl with 1.5 units of Platinum Taq polymerase (Amersham Biosciences, Uppsala, Sweden) and 10 µl of template. The following conditions were used: initial denaturation at 94°C for 5 min followed by 30 DNA denaturation cycles at 94°C for 1 min 30 s, 50°C for 1 min for primer annealing, and DNA synthesis elongation at 72°C for 4 min. A final elongation step was performed at 72°C for 7 min. PCR products were fractionated by 1% agarose gel electrophoresis, excised from the gel and purified using a GFX gel band purification kit (GE Healthcare Life Sciences, Uppsala, Sweden). Purified DNA was cloned using Clone Jet PCR kit (Promega, Madison, USA) to transform XL1 *Escherichia coli*. Recombinant clones were sequenced using automatic methods (AB13130 Applied Biosystems). Both strands were sequenced in all cases (Institute Pasteur de Montevideo). Sequences were analysed using Gene Runner software, and the translated ORFs were aligned to known FABPs using the CLUSTAL W2 software available at the EMBL-EBI website.

### Parasite material

The parasites (Mesocestoides vogae tetrathyridia) used in this research were provided by the Laboratory of Animal Experimentation (Facultad de Química, Universidad de la República, Montevideo, Uruguay). Tetrathyridia (Tt) were maintained by intraperitoneal passage through male CD1 mice (3 months old) and harvested by peritoneal aspiration and extensively washed with Hank’s balanced salt solution (Sigma-Aldrich, St. Louis, USA). Mice were sacrified by cervical dislocation. Anesthetics were avoided because these products may affect the biology of the parasite. Anyway cervical dislocation is a painless procedure and does not require anesthetic. Mice maintenance and infection were performed by the Laboratory of Animal Experimentation (Facultad de Química, Universidad de la República, Montevideo, Uruguay). The animal care committee of Uruguay, (CHEA) has approved the protocols for the cestode *Mesocestoides vogae* maintenance for the use of laboratories of our University and mice sacrifice proceedings. These were carried out following the guidelines of the Canadian Council on Animal Care.

### Production of recombinant proteins

Recombinant MvFABPa and MvFABPb were expressed in a BL21-pET-5a host/vector expression system (Promega, Madison, USA). To synthesise the MvFABPa and MvFABPb coding regions for expression in *E. coli,* sense and antisense oligonucleotides were designed and used to perform PCRs with reverse transcribed RNA from *M. vogae* as a template. Purified PCR fragments were cloned into the pET-5a vector. The constructs were transformed into *E. coli* BL21 (DE3) and used for expression of the recombinant proteins. Both strands were sequenced (Institut Pasteur de Montevideo).

Transformed bacteria were cultured overnight at 37°C in 2TY medium containing 100 µg ampicillin/ml. The culture was diluted 1/33 to 200 ml in the same medium, grown to A_600_ = 0.5 to 0.8 and induced with 0.45 mM isopropyl L-D thiogalactopyranoside at 37°C and 200 rpm. After 2 hrs of induction, the cells were harvested by centrifugation at 4600×g for 30 min and resuspended in 30 mM Tris-HCl, pH 8.3, 1 mM EDTA, and 1 mM DTT. Lysis was performed by three freeze-thaw cycles at −20°C and nine cycles of 30 s sonication with 30% amplitude (Branson Ultrasonic Corporation). The solution was clarified at 27200×g for 30 min at 4°C. The supernatant was concentrated by ultrafiltration and applied to a Sephadex G-50 (Pharmacia, Uppsala, Sweden) column (1.6 cm×1 m) equilibrated in 30 mM Tris-HCl, pH 8.3. The column was eluted with the same buffer with a flow rate of 15 ml/hr. Fractions containing the fatty acid binding protein were combined and subjected to anion exchange chromatography using Q-Sepharose (Sigma-Aldrich Co., St. Louis, USA) in batches and equilibrated in the buffer used previously. A step gradient of NaCl (25 mM, 35 mM, 45 mM, 100 mM, and 1 M NaCl) was used to elute the protein. Selected fractions were concentrated by ultrafiltration and protein concentration was determined by spectrophotometry. Molar extinction coefficients were calculated using Protoparam tool of Expasy platform (MvFABPa: ε_280_ = 9970 M^−1 ^cm^−1^ and MvFABPa: ε_280_ = 8480 M^−1 ^cm^−1^).

### Antibody purification

Polyclonal antisera were raised in New Zeland white rabbits by Polo Tecnológico de Pando (Facultad de Química, Universidad de la República, Montevideo, Uruguay). Serum against MvFABPa and MvFABPb were precipitated with ammonium sulphate according to Opperman [25]. Since antibodies share cross reaction, they were purified by affinity chromatography with cianogen bromide activated sepharose (Sigma-Aldrich, St. Louis, USA). Briefly, 0.5 g of cianogen bromide activated sepharose was mixed with 3 ml of HCl 1 mM during 2 hs, and afterwards washed with 50 ml of HCl 1 mM, 10 vol. of distilled water and 1.25 ml of binding buffer (0.1 M NaHCO_3_, 0.5 M NaCl, pH 8.3–8.5). The resin was incubated with the purified recombinant protein in the binding buffer during 2 hs at room temperature. After washing with binding buffer the remainder sites were blocked overnight with glycine 0.2 M pH 8 buffer, at 4°C. Five washing cycles were performed, first with binding buffer and afterwards with acetate 0.1 M pH 4, NaCl 0.5 M. Samples were loaded after columns equilibration with working buffer (0.1 M glycine, 0.15 M NaCl, pH 8.2). Finally, working buffer washes were performed until absorbance at 280 nm was equal to 0 and purified antibodies were eluted with elution buffer (0.1 M glycine, 0.5 M NaCl, pH 2.6). Immediately, 0.1 vol. of Tris-HCl 1 M pH 8.3 was added to the collected fractions to neutralize them. The specificity of the purified antibodies was tested using Western Blots assays.

### MvFABPs immunolocalization studies

Immunohistochemical studies were performed on criosections of larvae using purified polyclonal antibodies raised against MvFABPa and MvFABPb. To examine protein-ligand co-localization at cytoplasmic and nuclear level, *M. vogae* larvae were cultured in presence of the BODIPY FL C_16_. To analyze MvFABPs mitochondrial localization the larvae were cultured in presence of MitoTracker fluorescent marker.

Immediately after extraction from mice, Tt were cultured for five days in RPMI-1640 (Sigma-Aldrich, St. Louis, USA) and 10% foetal bovine serum, followed by one day of culture without serum, according to Britos and coworkers [24]. Some larvae were then incubated with 0.2 µM BODIPY FL C_16_ for 15 min at 37°C and others with 0.2 µM MitoTracker Orange CMTMRos for 15 min at 37°C. Fluorescent reagents were purchased from Invitrogen (California, USA). After treatment, live parasites were extensively rinsed with PBS. Then, Tt were fixed in 3% paraformaldehyde in PHEM buffer (25 mM Hepes, 60 mM Pipes, 10 mM EGTA, and 2 mM MgCl_2_), pH 7.5, for 60 min with orbital agitation at 4°C. Samples were extensively washed with PHEM buffer. Larvae were embedded in 30% sucrose in PHEM overnight at 4°C. Later, the samples were embedded in graded sacarosa/criopreservation media (Jung, Lieca Microsystems, Germany) and finally embedded in criopreservation media. Ten microns thick sections were used to immunostaining and confocal microscopy analysis. The sections were incubated during 30 min with a blocking solution (0.1% BSA, 150 mM Glycine, 5% normal goat serum in PHEM buffer) at room temperature, and 60 min at 37°C with each specific antibody diluted 1/200 in working solution (0.1% BSA, 150 mM Glycine in PHEM buffer). After three washes with the same solution, the sections were incubated with the secondary antibody (goat anti-rabbit IgG conjugated to Alexafluor 488 or 546; Invitrogen, California, USA) diluted 1/1000 in working solution for 45 min at 37°C. In some assays, DAPI (Invitrogen) diluted 1/1000 was included. After extensive washes with working solution and PHEM buffer samples were mounted in Prolong Gold antifade reagent (Invitrogen, California, USA) and maintained at 4°C until observation using a Olympus BX61 scanning laser confocal light microscope. Representative images of 6 to 10 Tt in each condition were taken. The images were recorded from a single focal plane of 1 µm thick.

### Subcellular fractionation

0.5 ml of *M. vogae* larvae were homogenized on ice in 2.5 ml of homogenization buffer (10 mM Tris-HCl pH 7.6, 10 mM EDTA, 250 mM sucrose, 5 µg/ml benzamidine, 5 µg/ml iodoacetamide, 1 mM PMSF). The homogenate was centrifuged at 300×g for 15 min to remove cell debris and whole cells. The supernatant was subjected to sequential centrifugation: a) centrifugation of the extract at 900×g for 30 min; b) centrifugation of the supernatant at 10,000×g for 30 min; c) ultracentrifugation of the supernatant at 105,000×g for 2 hours. The second, third and forth residues were considered as the nuclear, mitochondrial and microsomal fraction, respectively, while the remaining solution was the cytosolic fraction. The nuclear, mitochondrial, and microsomal fractions were washed with homogenization buffer. The enrichment of the subcellular fractions was monitored by measuring marker enzyme activity in each fraction. The activity of lactate dehydrogenase (cytosolic enzyme) [26], succinate dehydrogenase (mitochondrial enzyme) [27] and peroxidase (peroxisomal enzyme) [28] was determined. Enrichment of nuclear fractions was verified by epifluorescence microscopy using DAPI as nuclear marker.

The obtained subcellular enriched fractions were purified with PlusOne 2-D Clean -Up Kit (Amersham Biosciences, USA). They are then concentrated with Amicon Ultra-15 Centrifugal Filter Device using a Millipore membrane (cut off 10 kDa) or Vivaspin 500 (Sartorius Stedim, Germany, cut off 10 kDa), following the manufacturer’s instructions.

### 2D electrophoresis and mass spectrometry analysis

Protein extracts of each enriched fraction (20 µg) were analyzed by two-dimensional electrophoresis (2D) and mass spectrometry. Protein quantifications were performed using BCA kit (Sigma Aldrich Co., St. Lous USA). 2D were performed in the UByPA service of Institut Pasteur de Montevideo. Isoelectric focusing was performed using the IPGphor system (Amersham Biosciences) into 7 cm IPG strips, with a pH range of 3–10. Electrofocused strips were loaded on 15% polyacrylamide gel (10×10×0.1 cm), submitted to electrophoresis and stained with AgN0_3._ Gels were fixed for 1 hr (45% ethanol/10% acetic acid) and incubated with 0.02% sodium thiosulfate for 1 min. After three washes with deionized water, gels were incubated for 30 min in 0.15% AgN0_3_ solution. To visualize spots, gels were washed and incubated in a developer solution (2% sodium carbonate and 0.04% formaldehyde). When spots were visually detected a stop solution (30% acetic acid, 4% Tris-base) was immediately used to stop the development. Spots of expected mass and isoelectric point were excised for mass spectrometry identification in the UByPA service of Institut Pasteur de Montevideo. Peptide mass fingerprints were submitted to MASCOT (Matrix Science http://www.matrixscience.com/search_form_select.html) software. Criteria to protein identification include: MASCOT scores, sequence coverage, molecular mass and isoelectric points of MvFABPs.

### Homology modelling

Homology modeling of MvFABPa and MvFABPb was performed by means of an alignment of sequences over that of FABP4 (Protein Data Bank 2Q95) and replacing all new side chains, keeping the main chain of template. The alignment was performed using for the initial pairwise build-in up a BLOSUM62 substitution matrix; Gap start = 7 and a gap extend = 1. Then a build-up tree based was performed with an iteration limit of 100 and a failure limit of 10 was used. Finally, for the structure alignment, a gap start of 1 and a gap extend of 0.1 were employed. Each model was submitted to an energy minimization in solvated phase and the force field Amber 99 [29]–[30]. Further 10 nanoseconds molecular dynamics simulation using periodic conditions were performed to test their stability. In the case of MvFABPb, a second model was generated through torsion of Cα-Cβ-Cγ-Cδ side chain atoms to reach the same configuration as in FABP4 (named from here “folded”). Three additional molecular dynamics simulations of 1 nanosecond length and further energy minimization were used to compare the behaviour of the lysine residue in the “extended” and “folded” configuration. The potential energy of this residue was calculated and compared with that of FABP4. All calculations were done running the Molecular Operating Environment (MOE 2007.09) suite on Linux, on a workstation with a quad-core processor Hyperthread equipped [31].

## Results

### Antibodies purification

**Figure 1:**
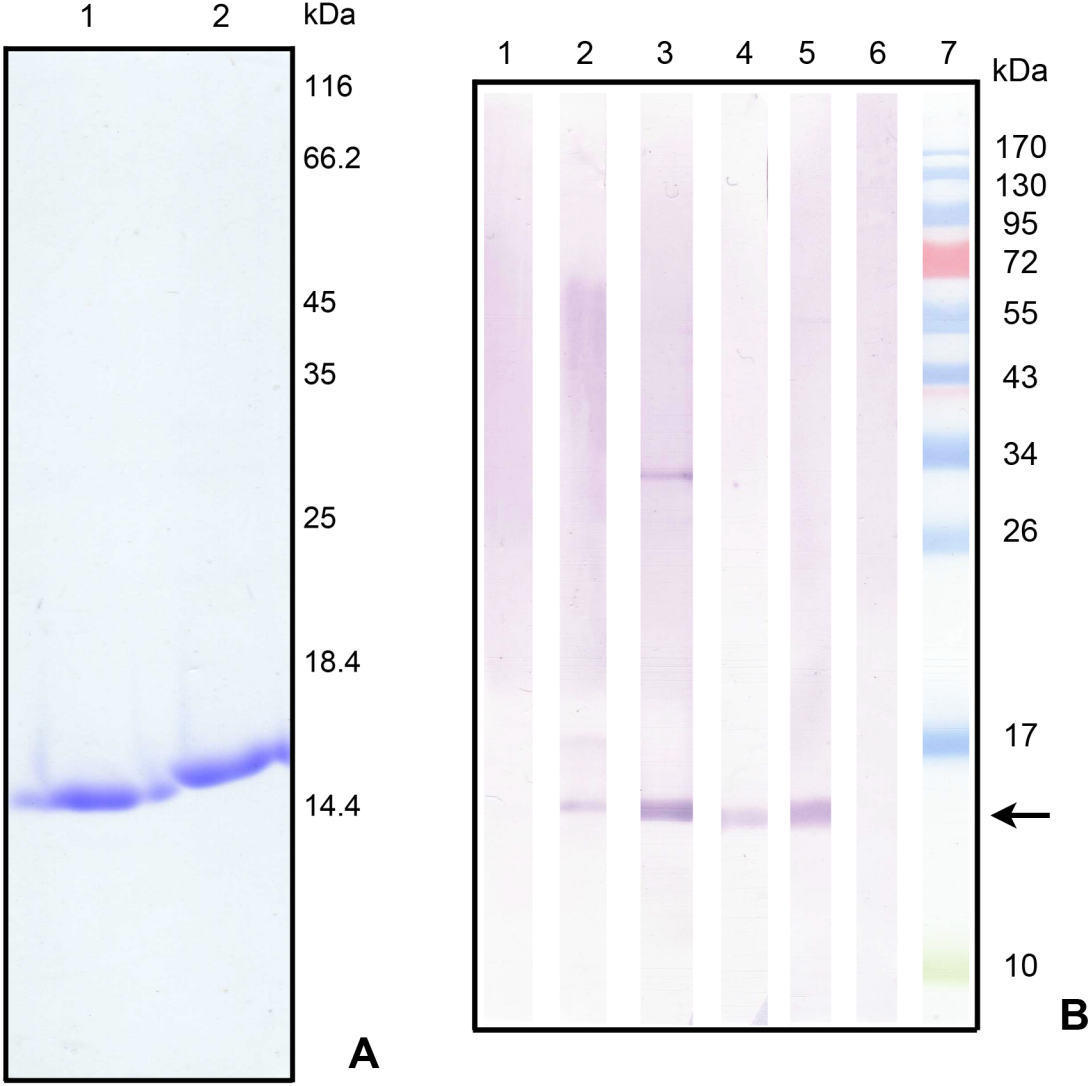
Purification of MvFABPa and MvFABPb recombinant proteins and the respective antisera. A) 15% SDS-PAGE with Coomassie brilliant blue staining: 1) 7 µg of purified MvFABPa; 2) 7 µg of purified MvFABPb. B) Western blot using purified antibody against MvFABPa (1, 2, 3) or against MvFABPb (4, 5, 6): 1 and 4) 2 µg of purified MvFABPb, 2 and 5) 30 µg of Tt protein extract, 3 and 6) 2 µg of partially purified MvFABPa; 7) Page Ruler Prestained Protein Ladder (SM0671, Fermentas, Thermo Fisher Scientific). Arrows indicate MvFABPa and MvFABPb.

In order to generate specific antibodies against MvFABPa and MvFABPb, the recombinant proteins were produced in *E. coli* BL21 (DE3) strain. The 5′ missing coding sequence of *Mvfabpa* and *Mvfabpb* genes were obtained using inverse polymerase chain reaction and the corresponding coding regions were cloned (GenBank GI:209977648, GI:443611292). The molecular masses derived from the complete sequences were 14844.0 Da and 14726.8 Da for MvFABPa and MvFABPb, respectively, and the isoelectric points were 5.54 for MvFABPa and 6.91 for MvFABPb. Purified recombinant proteins are shown in Figure 1A. The absence of cross-reactivity of the purified antibodies was checked by Western blot (Figure 1B). The 32 kDa band observed on line 3 corresponds to an *E. coli* protein undetected in the purified protein sample used as antigen (Figure 1A).

### Fatty acids uptake

**Figure 2:**
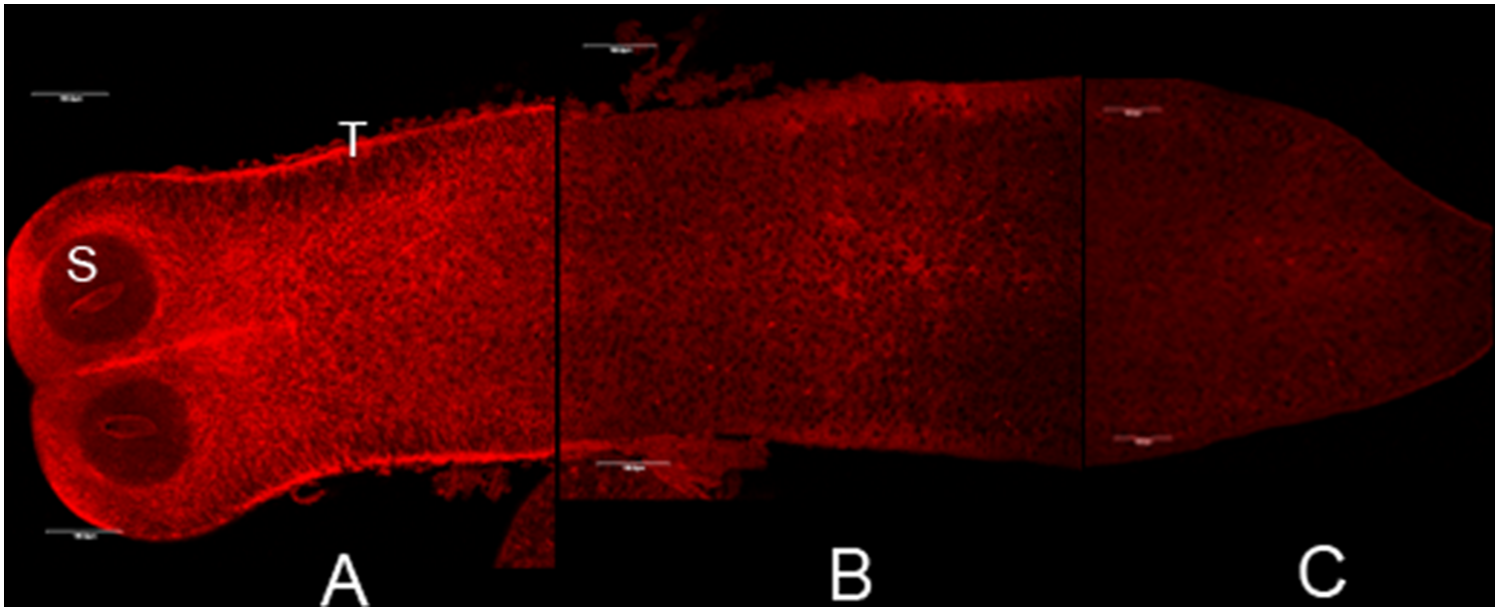
Tetrathyridia *in vivo* uptake of a fatty acid analogue BODIPY FL C_16_ along the larvae. A) Apical region; B) midregion; C) caudal region. Magnification 20X, bars indicate 10 microns.

The larval capacity to take up fatty acids *in vivo* was assayed adding BODIPY FLC_16_ to the culture medium_._ A strong signal at tegumental level of the apical region and at the parenchyma surrounding the suckers is appreciated in Figure 2A. A faint labelling was widely distributed in the parenchyma decreasing along the longitudinal larvae axis (Figure 2B, 2C). These data indicate that the FA uptake activity is higher at the apical region of the larvae. Thin superficial cytoplasm covering the suckers and short micotriches in the apical region could facilitate the capture of this FA analogue [32]. This region is also an important proliferative zone, particularly during growth phase [32]. It would not surprise us that FAs were specifically required in this area where the formation of new structures take place.

### Subcellular localization: immunohistochemical studies

Since parasitic Platyhelminthes depend on lipids uptake from the host, an efficient mechanism of transport between cells and intracellular compartments should exist. To investigate the putative role of MvFABPs in FA cytoplasmic-nuclear targeting, we cultured Tt in presence of the fluorescent FA analogue BODIPY FLC_16_. Previous works have shown that FABPs can bind this FA analogue [33], [34]. Confocal immunomicroscopy was then performed on cultured Tt larvae sections using MvFABPa and MvFABPb specific antibodies and DAPI as a nuclear marker.

**Figure 3:**
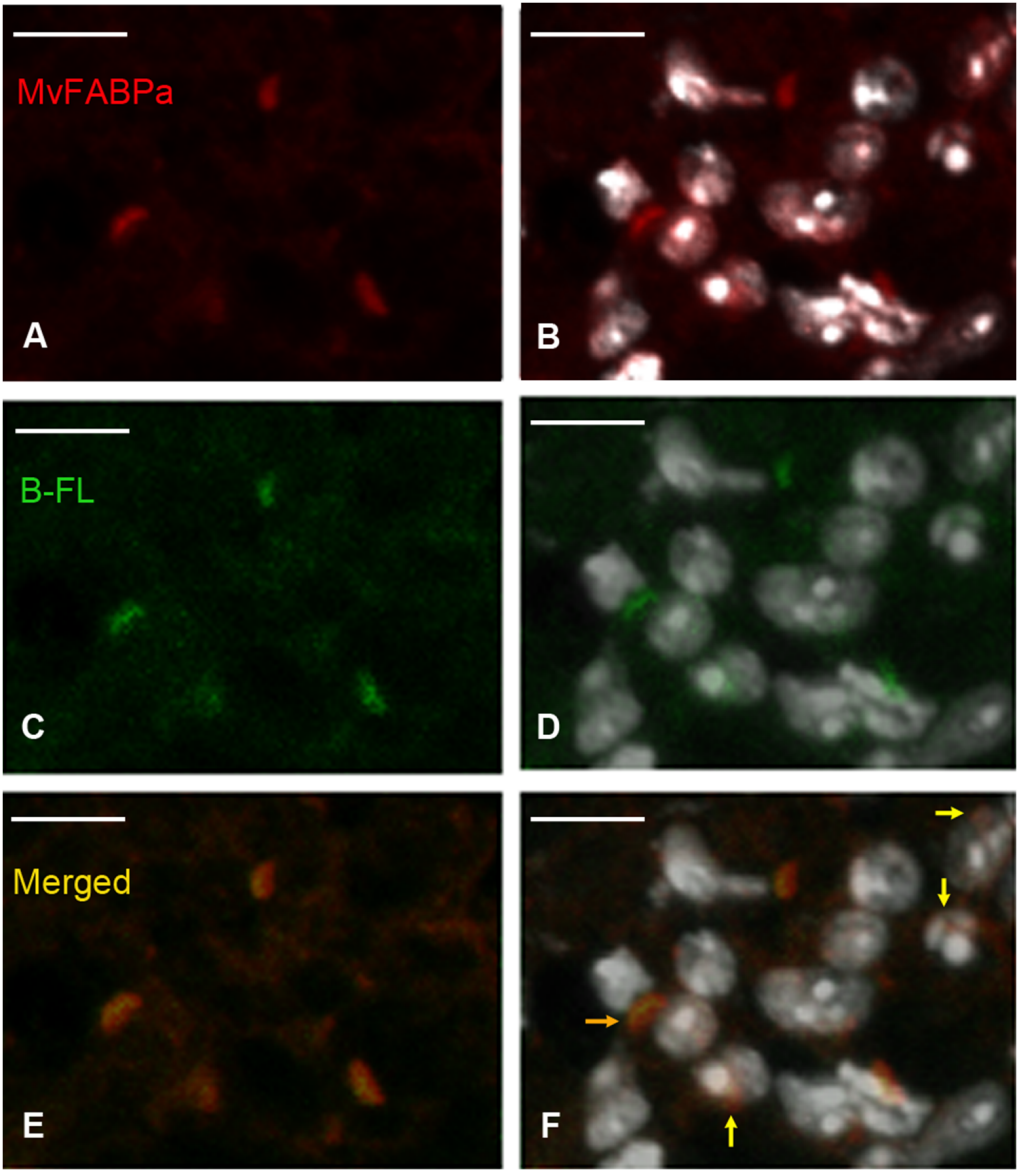
Tetrathyridia immunohistochemistry with antibody against MvFABPa, MvFABPa-nucleus-fatty acid analogue co-localization. A) MvFABPa (red), B) MvFABPa-nucleus (white, DAPI) merge, C) BODIPY FL C_16_ (green), D) BODIPY FL C_16_-nucleus merge, E) MvFABPa-BODIPY FL C_16_ merge (orange-yellow), F) MvFABPa-BODIPY FL C_16_–nucleus merge. Magnification 150X, bars indicate 5 microns. Yellow arrows show some MvFABPa-fatty acid analogue-nucleus co-localization points. The orange arrow indicates an intense MvFABPa- BODIPY FL C_16_ perinuclear co-localization signal. Bright white signals within the nuclei correspond to nucleoli.

**Figure 4:**
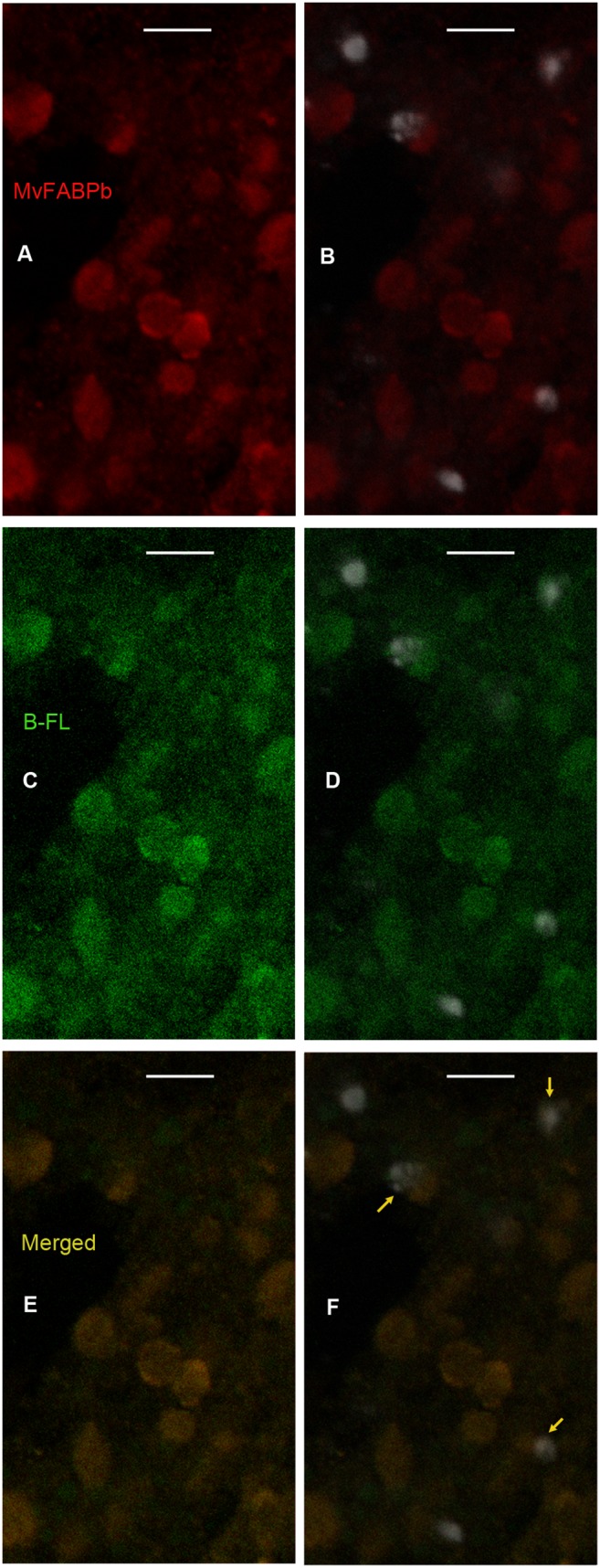
Tetrathyridia immunohistochemistry with antibody against MvFABPb, MvFABPb-nucleus-fatty acid analogue co-localization. A) MvFABPb (red), B)MvFABPb-nucleus (white, DAPI) merge, C) BODIPY FL C_16_ (green), D) BODIPY FL C_16_-nucleus merge, E) MvFABPb-BODIPY FL C_16_ merge (orange-yellow), F) MvFABPb-BODIPY FL C_16_–nucleus merge. Magnification 75X, bars indicate 10 microns. Yellow arrows show some MvFABPb-fatty acid analogue-nucleus co-localization regions.

Cytoplasmic and nuclear co-localization of BODIPY FLC_16_ with MvFABPs is depicted in Figures 3, 4 and Figure S1, showing that BODIPY FL C_16_ is targeted to the nuclei. We also emphasize that signals corresponding to MvFABPa are observed in most nuclei as well as forming accumulations in perinuclear regions (Figure 3B). This latter localization resembles that of endoplasmic reticulum localization. A broad cytoplasmic distribution of MvFABPs was also observed (Figure 3B, 4B). A rounded MvFABPb-FA co-localization labelling could be attributed to lipid droplets (Figure 4E, 4F, and Figure S1).

**Figure 5:**
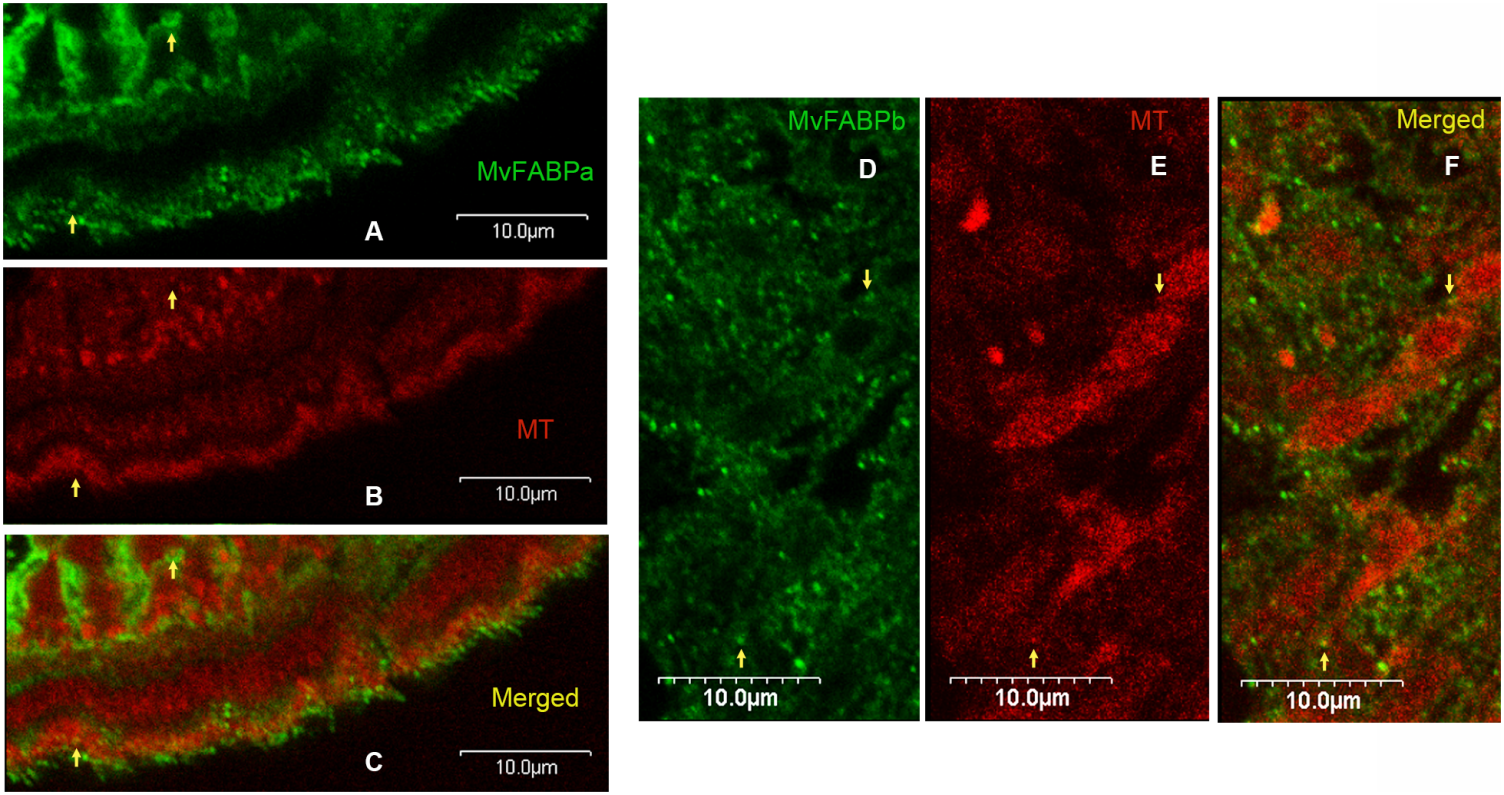
Tetrathyridia immunohistochemistry, MvFABP-mitochondria co-localization. A) MvFABPa (green), B) mitochondria (red, Mitotracker), C) MvFABPa-mitochondria merge. A–C: magnification 250X, bars represent 10 microns. D) MvFABPb (green), E) mitochondria (red, Mitotracker), F) MvFABPb-mitochondria merge. D–F: magnification 300X, bars represent 10 microns. Yellow arrows indicate some MvFABP-mitochondria co-localization points.

Using mitochondria-specific fluorescent probe we demonstrated that both MvFABPs co-localizes with this organelle (Figure 5). This observation is unexpected since Platyhelminthes have a restricted FA oxidation [20]. Controls of all immunohistochemical experiments are shown as supplementary material (Figures S2 and S3).

### Subcellular localization: mass spectrometry identification

**Figure 6:**
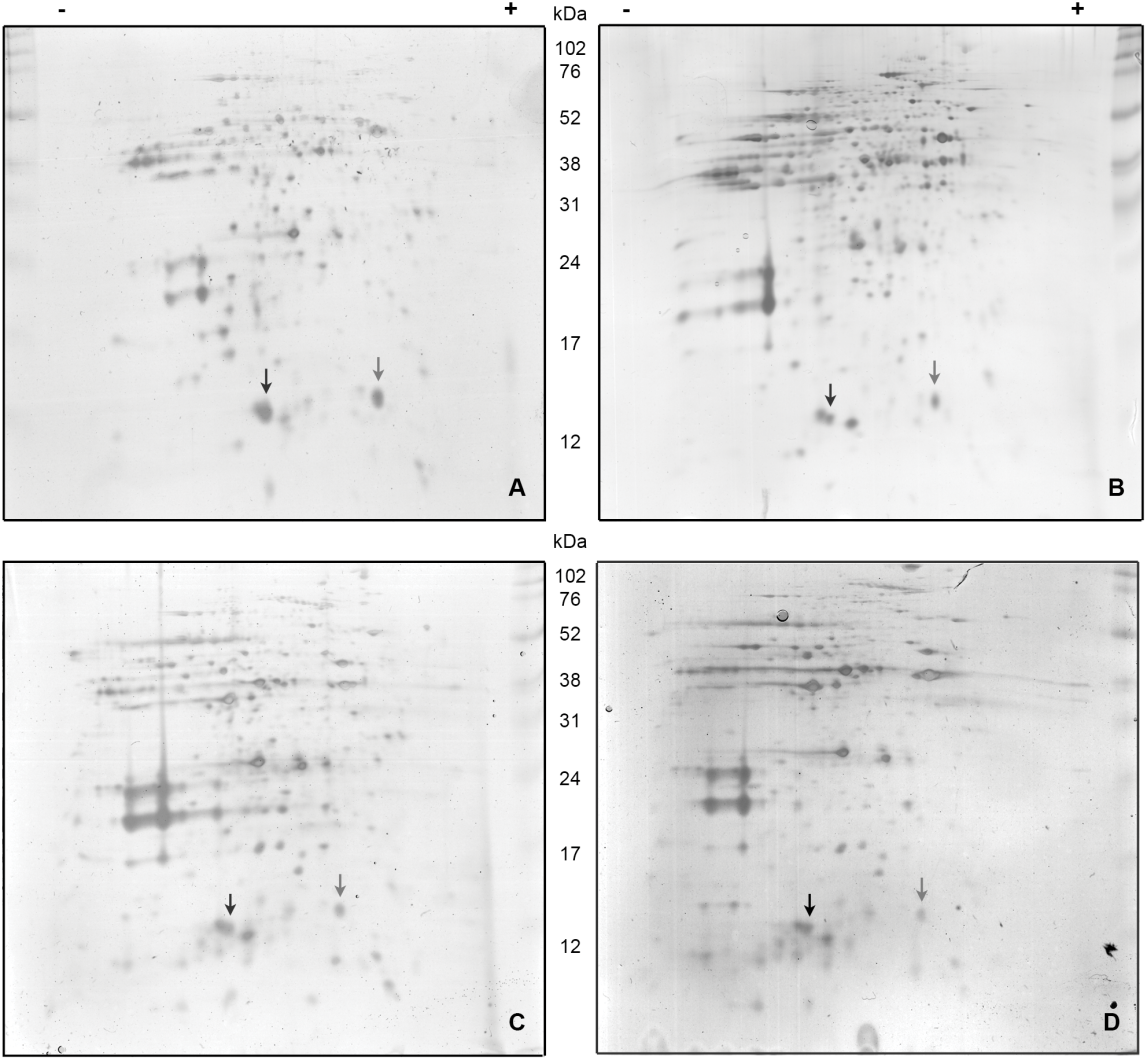
Electrophoresis 2D-mass spectrometry identification of MvFABPs in tetrathyridia enriched subcellular fractions. A) Enriched cytosolic fraction; B) enriched nuclear fraction; C) enriched mitochondrial fraction; D) enriched microsomal fraction. Full Range Rainbow Molecular Weight Marker (Amersham, GEHealthcare). Black and gray arrows indicate MvFABPb and MvFABPa, respectively.

To verify immunolocalization results we performed mass spectrometry MALDI/TOF-TOF analysis of nuclear, mithocondrial, microsomal and cytoplasmic larvae enriched fractions obtained by differential centrifugation. Fractions enrichment is shown in Table 1 and Figure S4. Proteins of each enriched fraction were separated by 2D electrophoresis and those spots of the expected molecular mass and isoelectric point were removed and analysed (Figure 6). The obtained mass spectra submitted to MASCOT software indicated that identification of both MvFABPs was statistically significant in all the enriched fractions studied, with a coverage >40%. Identification of MvFABPa in microsomal enriched fraction showed a lower sequence coverage (20%) but statistically significant as well. MvFABPs mass spectra of cytosolic enriched fraction are shown as supplementary material (Figure S5).

**Table 1:** Purity of enriched fractions.

*Lactate Dehydrogenase*	Enz. act. (U/ml)	[Prot] (mg/ml)	Sp. act. (U/mg)	Purific. f.
Homogenate	0.043±0.003	1.907	0.023±0.002	1.00±0.09
Nuclear fraction	0	0.739	0	0
Mitochondrial fraction	0	2.323	0	0
Microsomal fraction	0	3.843	0	0
Cytosolic fraction	0.053±0.003	0.723	0.073±0.004	3.20±0.18
***Succinate Dehydrogenase***	**Enz. act. (U/ml)**	**[Prot] (mg/ml)**	**Sp. act. (U/mg)**	**Purific. f.**
Homogenate	0.0068±0.0010	1.907	0.0036±0.0005	1.00±0.14
Nuclear fraction	0	0.739	0	0
Mitochondrial fraction	0.0283±0.0019	2.323	0.0122±0.0008	3.39±0.22
Microsomal fraction	0.0010±0.0015	3.843	0.0003±0.0004	0.08±0.11
Cytosolic fraction	0	0.723	0	0
***Peroxidase***	**Enz. act. (U/ml)**	**[Prot] (mg/ml)**	**Sp. act. (U/mg)**	**Purific. f.**
Homogenate	3.89±0.45	1.907	2.04±0.24	1.00±0.12
Nuclear fraction	0	0	0	0
Mitochondrial fraction	0.45±1.03	2.323	0.19±0.44	0.09±0.21
Microsomal fraction	13.53±0.34	3.843	3.52±0.09	1.73±0.04
Cytosolic fraction	0.22±0.22	0.723	0.30±0.30	0.15±0.15

The enzymatic activity (Enz. act.), specific activity (Sp. act.) and the purification factor (Purif. f.) are shown with the standard deviations. [Prot]: protein concentration.

### *In silico* localization signals study

**Figure 7:**
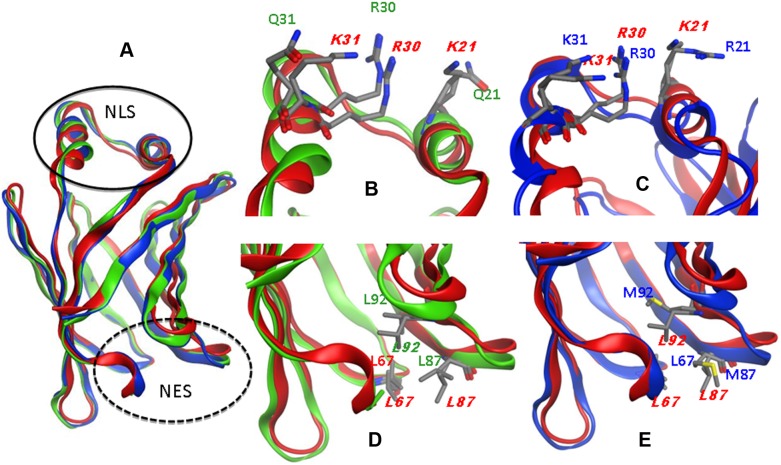
NLS and NES signals. Superimposed models of FABP4 and MvFABPs;FABP4 (red); MVFABPa (green); MvFABPb (blue). A) FABP4-MvFABPa-MvFABPb; B–C) NLS region; D–E) NES region.

We searched for tridimensional nuclear localization signals (NLS) and nuclear exportation signals (NES) reported for other FABPs [35] employing homology models. The obtained MvFABP models contain the β-barrel tertiary structure typical of FA-binding protein family members (Figure 7A). The global root mean square deviations (RMSD) between these MvFABPa and MvFABPb models and their template were 0.94 Å and 0,98 Å respectively. Upon graphical inspection, the *in silico* and crystallographic models appear similar. To verify the stability and integrity of the modelled structures, the total potential energy evolution during the 10 nanosecond molecular dynamics simulations was registered and found stable after an equilibration time of 1 nanosecond in both protein models.

Superimposition of each MvFABP structure over FABP4 protein showed that the configuration of residues R_21_, R_30_ and K_31_ of MvFABPb and L_67_, L_87_ and L_92_ of MvFABPa was similar to FABP4 NLS and NES signals respectivelly (Figure 7). An extended position of K_31_ of MvFABPb obtained in the first steps of modeling, was particularly observed (Figure 7C). Two models were generated: a) one of them with the side chain of K_31_ in the mentioned extended configuration and b) the other one through a manual torsion of Cα-Cβ-Cγ-Cδ side chain atoms to reach the same configuration as in FABP4 (named from here “folded”). After molecular dynamics simulations the potential energy of this residue was −116.207 (FABP4), -106.659 (MvFABPb folded) and −74.852 (MvFABPb extended) kcal/mol, indicating a clear preference for the “folded” configuration. Moreover, the electrostatic surface potential also supports th hypothesis that the protein could have the NLS signal. Moreover, MvFABPa could not have a NLS because the Q-R-Q pattern has a very low positive charge with respect to the previously observed in FABP4 and in MvFABPb proteins.

## Discussion

Platyhelmithes do not synthesize *de novo* their own FAs. Low fatty acid aqueous solubility would strongly imply that specific and efficient mechanisms must exist to transport and target these compounds between and within cells. MvFABPs are good candidates to participate in *M. vogae* Tt FA intracellular distribution acting as counterparts of other lipid binding proteins involved in shuttling FAs to the surrounding host tissue [36]. In the present studies we investigate the intracellular distribution of MvFABPa and MvFABPb and the co-localization with fluorescent FA *in vivo* captured.

It has been demonstrated that members of the FABP family could be implicated in metabolic pathways related to the tissue(s) in which they are expressed. Several members of the family have been localised in the nuclei, mitochondria and Golgi of cultured cells [33], [37]–[38]. Mitochondrial localization of MvFABPs could be indicative of a relationship with the oxidation processes. This result is rather unexpected because the β-oxidation pathway is considered to be inactive in these parasites [39]. However, it has been reported that many platyhelminthes express β-oxidation enzymes, many of them with high activity, particularly acetyl-CoA acyltransferase [40]–[41]. Recently, Vinaud and co-workers showed that *Taenia crassiceps* is capable of producing energy from lipids as an alternative energy source in the case of glycogen or glucose shortage [42]. It is worth mentioning that the experimental conditions of the present work included an energetic source depletion. Considering this data, we cannot discard the hypothesis that FABPs could be a source of FAs for mitochondrial energetic metabolism in *M. vogae* under glucose deprivation.

Both MvFABPs were also detected in the enriched microsomal fraction. This fraction comprises small heterogeneous vesicles composed by endoplasmic reticulum, Golgi, plasma membrane, peroxisomes and lysosomes. This complexity makes difficult to atribute a putative role to these proteins.

The studies using a DNA marker indicates that both *M. vogae* FABPs as well as BODIPY FL C_16_ are directed to the nuclei of larvae cells. Moreover, MvFABPb has the putative 3D nuclear localization signal (R_21_, R_30_, K_31_) in frame with that of FABP4 [17], [35]. The classical NLS is a triad of basic aminoacids identifiable in the primary sequence of a protein [43]. The primary sequence of many members of FABPs family does not harbor a readily identifiable nuclear localization signal (NLS). However, such a signal could be found in the three-dimensional structure of mouse FABP4 mapped to three basic residues (K_21_,R_30_,K_31_) that form a functional NLS stabilized upon ligand binding [17], [35]. Such signal is recognized by adaptor proteins known as alpha importins [43]. Proteins of this family were identified in platyhelminth parasites, including the cestode *E. granulosus* (GenBank: GI 576700461/GI 576695743). In addition, MvFABPa has a putative nuclear export signal (NES) composed by three protuding Leucines: Leu67, Leu87, and Leu92. Similarly to the 3D NLS, a classical NES (L-X2-3L-X2-3L-X-L) [44] is not apparent in the primary sequence, but can be identified in the protein’s 3-dimensional fold. This signal is present in FABP4 whose exportation from the nucleus is mediated by CMR1 export machinery [35].

Despite the structural and nuclear/cytosolic localization similarities of MvFABPb and MvFABPa, the former could be activelly imported to the nucleus while the latter exported from the nucleus by mechanisms related to NLS and NES signals, respectivelly. CRABP-II and L-FABP, have cytosolic and nuclear localizations but lack one or two of the mentioned signals. In this sense, different types of movilization mechanisms were suggested [35], [45]. Therefore, a putative function of *M. voage* FABPs could be related to nuclear/cytosolic FA trafficking. In this scenario, MvFABPb could be involved in the entrance of FA linked to alpha importins or similar partners, while MvFABPa could export FA in a CMR1-like dependent pathway.

These proteins could be involved in nuclear lipids biosynthesis, lipid droplets formation, or gene expresssion regulation. Nuclear phospholipid byosinthetic pathways have been reported [46]–[49]. Recently, lipid droplets domains were identified in the nucleus where nuclear neutral lipids are stored. [50]. A role of FABPs as regulators of gene expression was already proposed [51]. FAs are activating ligands for nuclear receptors (such as PPARs, HNF-4α, LXR, and SREBP) and act as regulators of gene expression [51]–[52]. Various experimental approaches have shown that members of the FABP family are targeted to the nuclei and are detectable inside the nucleoplasm [53]–[55]. Moreover, it has been demonstrated that L-FABP-FA, K-FABP-FA and A-FABP-FA activate PPARα, PPARb and PPARg by direct interaction with these transcription factors, respectively [37], [55]–[57]. In this context, we cannot discard a role of MvFABPs in gene expression regulation. Members of the nuclear receptor family have been identified in parasitic Platyhelmithes, including Cestoda (www.genedb.org).

Our results extend the canonical role of taenia FABPs as simple cytoplasmic FA carriers. The ancestral FABP could play roles related to FA oxidation and gene expression regulation. Although various FABPs types have been identified in vertebrates, almost all invertebrate FABPs have high pairwise sequence identity with the same type of FABP [10]. This reduced diversity in invertebrates, in combination with our localization results, suggests that these molecules have a larger repertoire of interactions in the cell than vertebrate FABPs. Therefore, it is likely that the ancestral FABP could satisfy all the functions of the actual vertebrate’s proteins of the family. Functional specialization could have been the result of subtle changes in the internal cavity or on the surface that favoured interactions with specific targets.

Future work will be performed to describe with more accuracy the localization in each cellular compartment as well as to identify putative molecular partners.

## Supporting Information

Figure S1 **Tetrathyridia immunohistochemistry with antibody against MvFABPb, MvFABPb-nucleus-fatty acid analogue co-localization.** A and D) nucleus (white, DAPI); B and G) MvFABPb (red); E and H) BODIPY FL C_16_ (green); C) MvFABPb-nucleus merge (pink); F) MvFABPb-nucleus merge (light green); I) MvFABPb-BODIPY FL C_16_ merge (yellow). Magnification 150X, bars indicate 5 microns. Yellow arrows in C and F indicate a MvFABPb-nucleus-fatty acid analogue colocalization. The yellow arrow in I shows a round structure with MvFABPb-BODIPY FL C_16_ colocalization.
(TIF)

Figure S2 **Tetrathyridia immunohistochemistry controls.** A–D (magnification 150X): purified rabbit normal serum and Alexa Fluor 488 Goat anti-Rabbit IgGs antibody (A y B) or Alexa Fluor 546 Goat anti-Rabbit IgGs antibody (C y D). A and C: UV laser (DAPI), B: 488 nm laser, D: 543 nm laser. E and F (magnification 60X): autofluorescence (without antibodies); E: 488 nm laser, F: 543 nm laser.
(TIF)

Figure S3 **Tetrathyridia immunohistochemistry controls.** A–D: antibody against MvFABPa, without anti-Rabbit IgGs antibody; E–H: antibody against MvFABPb without anti-Rabbit IgGs antibody. A and E: 488 nm laser; B y F: 543 nm laser; C and G: UV laser; D and H: three channels merge. Magnification 150X.
(TIF)

Figure S4 **Epifluorescence micrograph of tetrathyridia nuclear fraction stained with DAPI.** Magnification 100X, the bar corresponds to 5 microns.
(TIF)

Figure S5 **MvFABPs mass spectrometry identification of tetrathyridia cytosolic enriched fractions.** A) MvFABPa identification B) MvFABPb identification. Top: mass spectrum; botton: list of peptide masses.
(DOCX)
