# Supplementary figures and images for: Towards an Understanding of Mesocestoides vogae Fatty Acid Binding Proteins’ Roles
Source: PLoS One. 2014 Oct 27;9(10):e111204. doi: 10.1371/journal.pone.0111204 (PMC4210247; doi:10.1371/journal.pone.0111204)

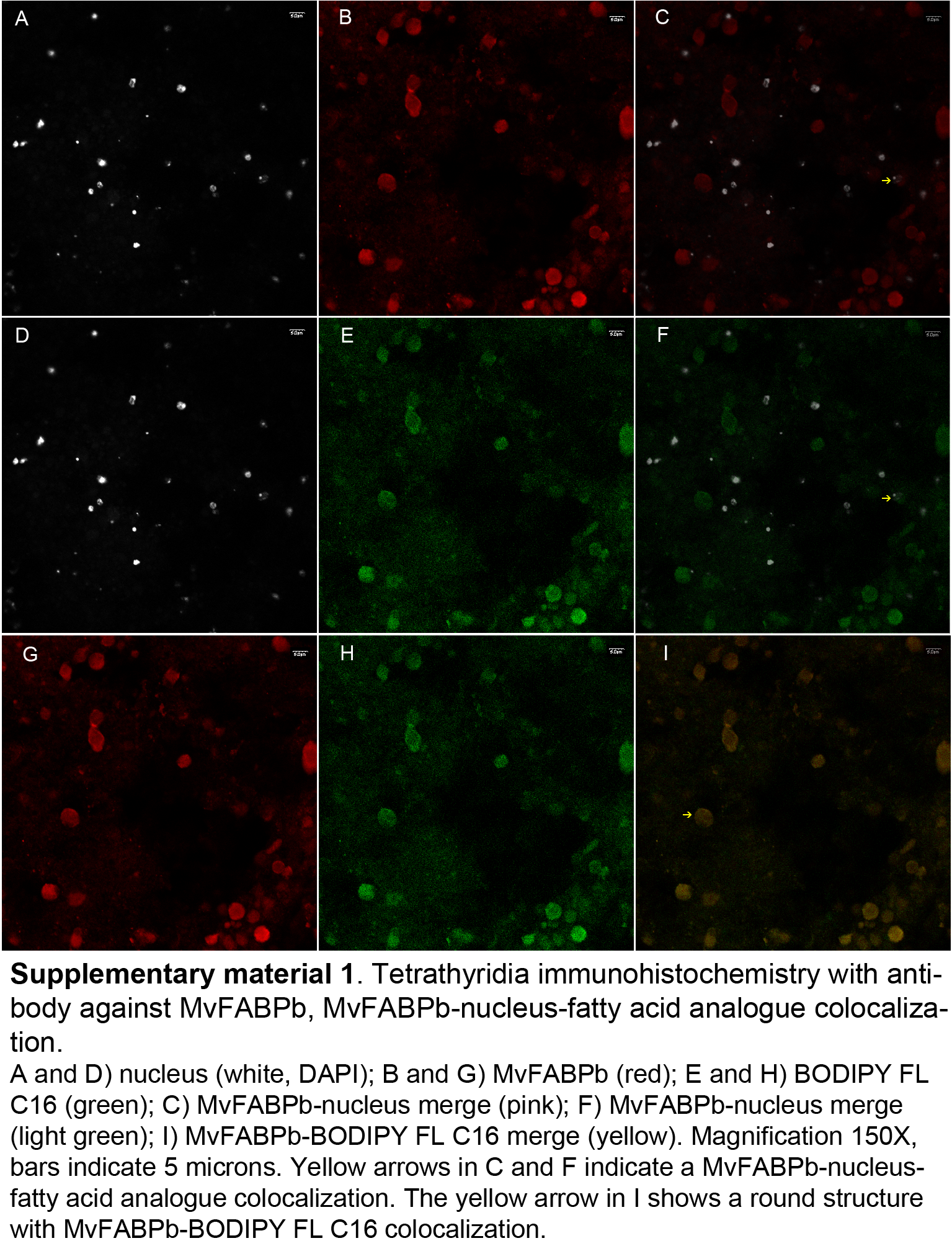

Supplement: Figure S1 — Tetrathyridia immunohistochemistry with antibody against MvFABPb, MvFABPb-nucleus-fatty acid analogue co-localization. A and D) nucleus (white, DAPI); B and G) MvFABPb (red); E and H) BODIPY FL C16 (green); C) MvFABPb-nucleus merge (pink); F) MvFABPb-nucleus merge (light green); I) MvFABPb-BODIPY FL C16 merge (yellow). Magnification 150X, bars indicate 5 microns. Yellow arrows in C and F indicate a MvFABPb-nucleus-fatty acid analogue colocalization. The yellow arrow in I shows a round structure with MvFABPb-BODIPY FL C16 colocalization. (TIF) [file pone.0111204.s001.tif]

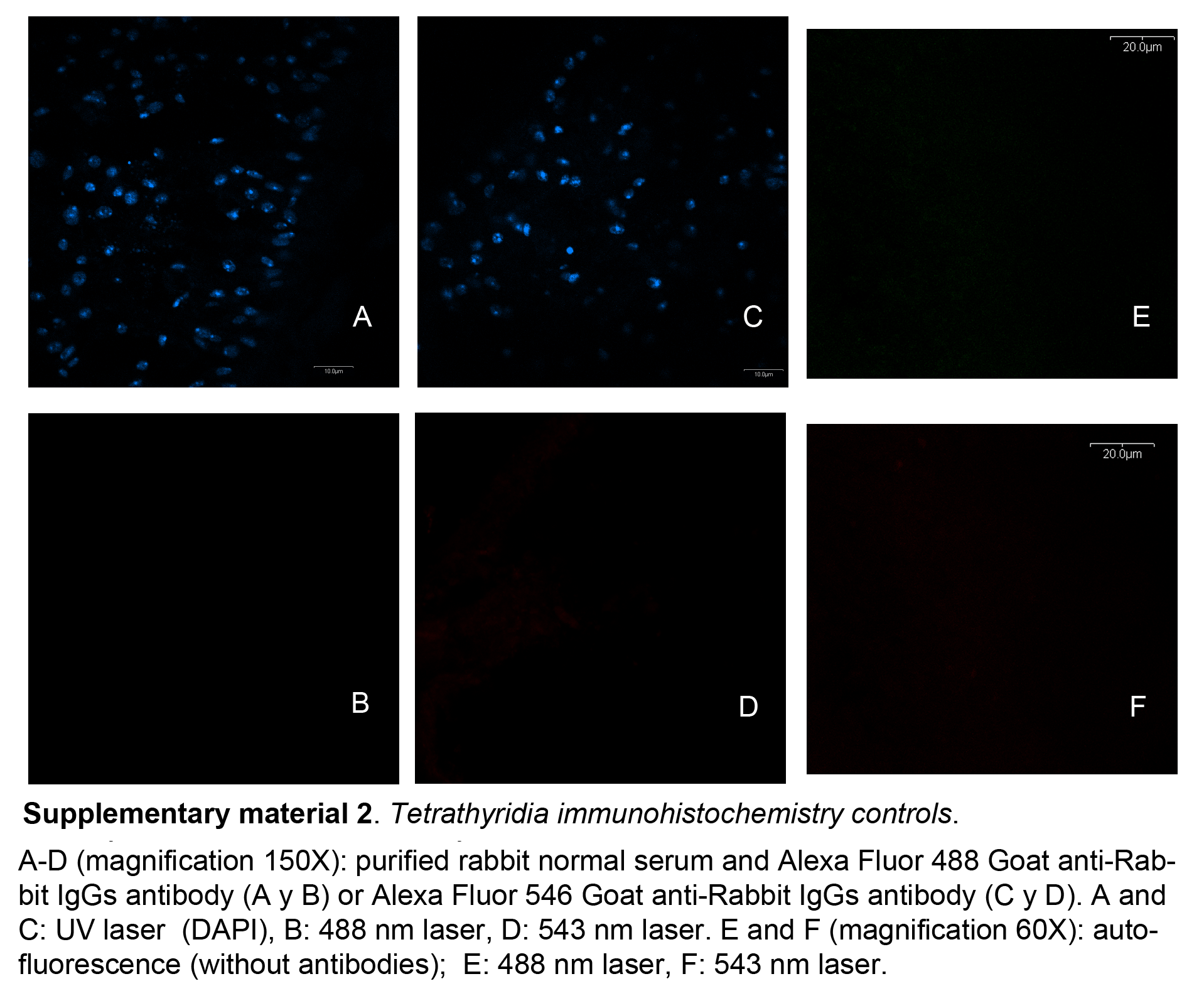

Supplement: Figure S2 — Tetrathyridia immunohistochemistry controls. A–D (magnification 150X): purified rabbit normal serum and Alexa Fluor 488 Goat anti-Rabbit IgGs antibody (A y B) or Alexa Fluor 546 Goat anti-Rabbit IgGs antibody (C y D). A and C: UV laser (DAPI), B: 488 nm laser, D: 543 nm laser. E and F (magnification 60X): autofluorescence (without antibodies); E: 488 nm laser, F: 543 nm laser. (TIF) [file pone.0111204.s002.tif]

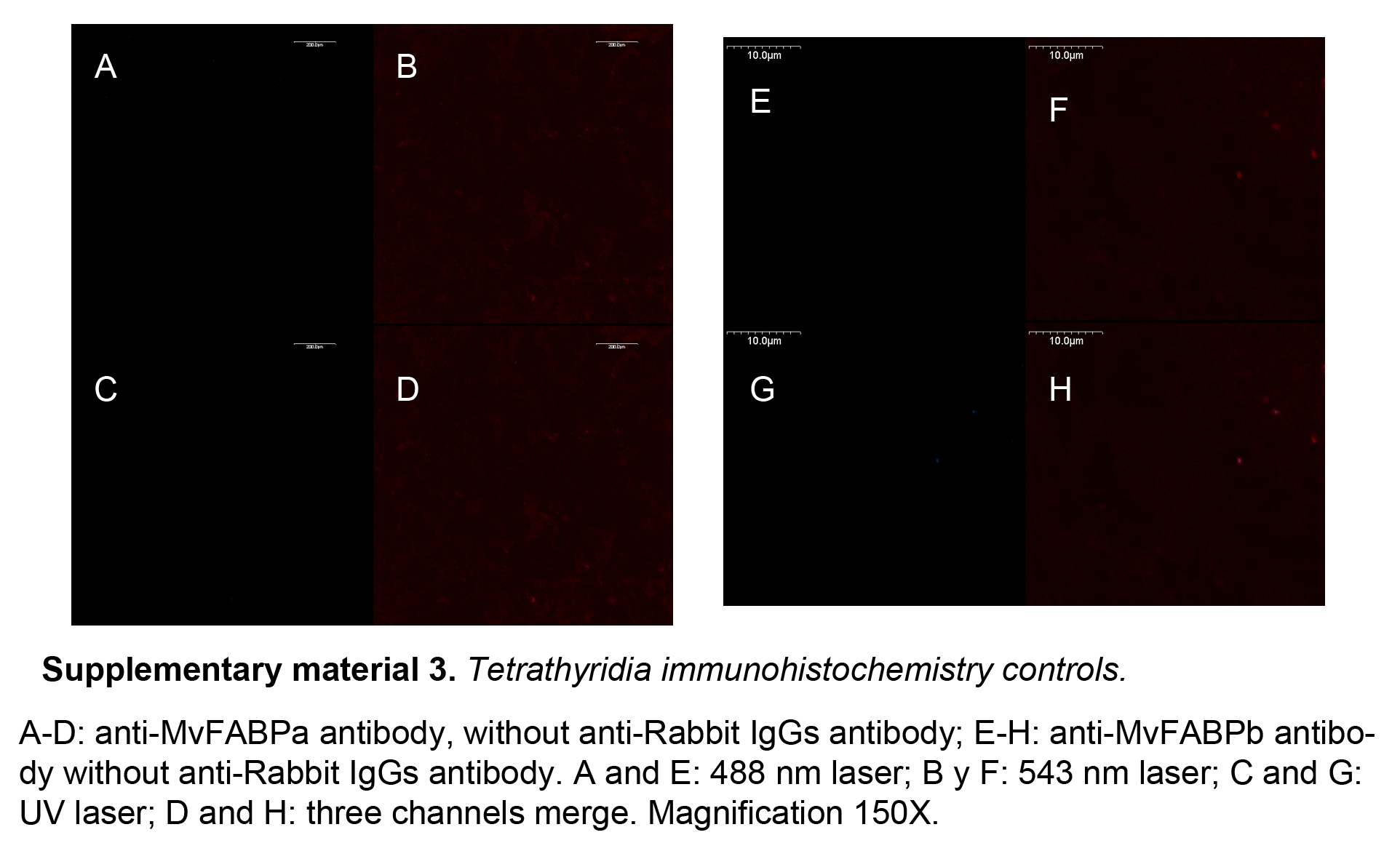

Supplement: Figure S3 — Tetrathyridia immunohistochemistry controls. A–D: antibody against MvFABPa, without anti-Rabbit IgGs antibody; E–H: antibody against MvFABPb without anti-Rabbit IgGs antibody. A and E: 488 nm laser; B y F: 543 nm laser; C and G: UV laser; D and H: three channels merge. Magnification 150X. (TIF) [file pone.0111204.s003.tif]

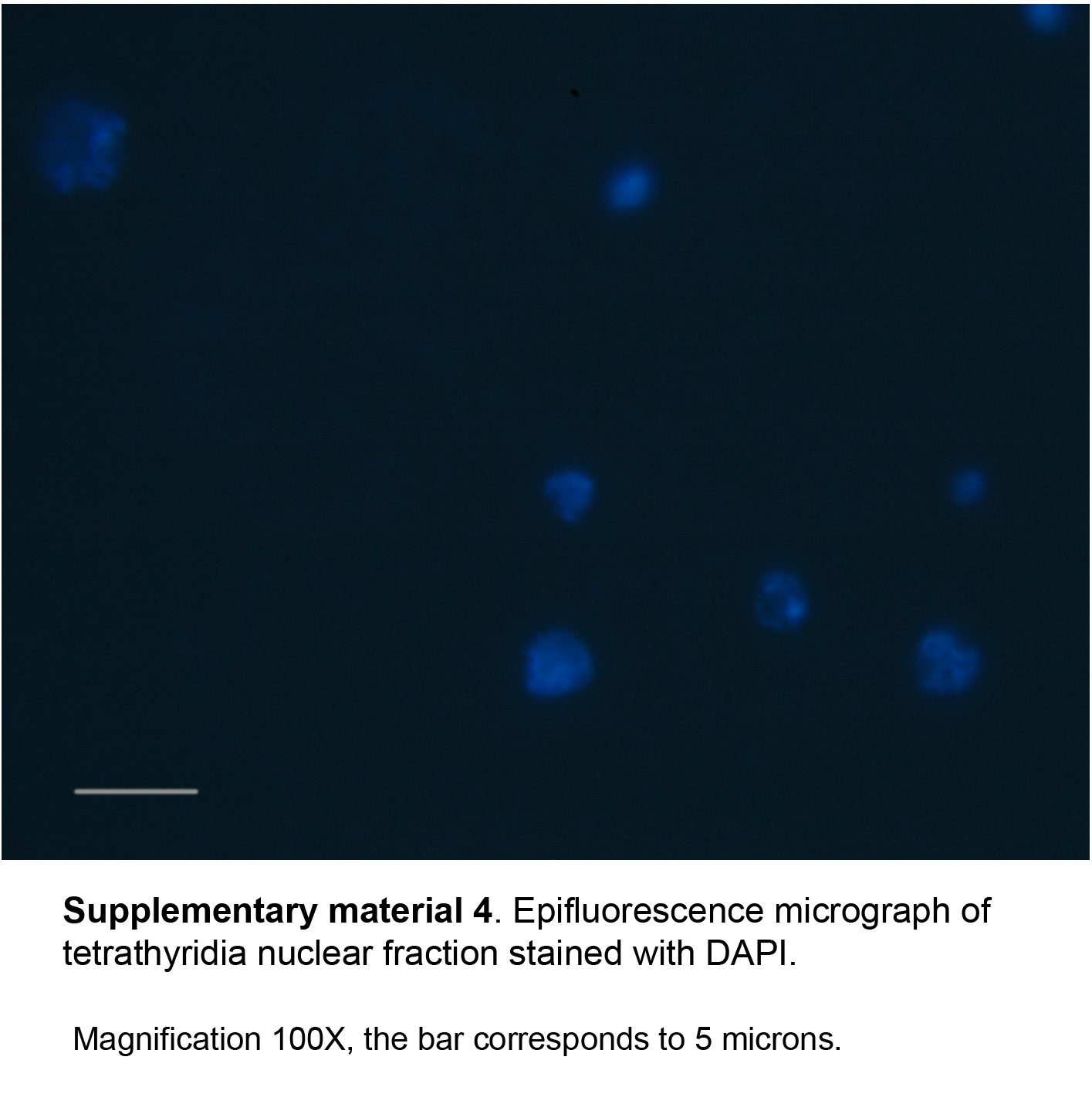

Supplement: Figure S4 — Epifluorescence micrograph of tetrathyridia nuclear fraction stained with DAPI. Magnification 100X, the bar corresponds to 5 microns. (TIF) [file pone.0111204.s004.tif]
