# Supplementary material for: Towards an Understanding of Mesocestoides vogae Fatty Acid Binding Proteins’ Roles
Source: PLoS One. 2014 Oct 27;9(10):e111204. doi: 10.1371/journal.pone.0111204 (PMC4210247; doi:10.1371/journal.pone.0111204)
Supplement: Figure S5 — MvFABPs mass spectrometry identification of tetrathyridia cytosolic enriched fractions. A) MvFABPa identification B) MvFABPb identification. Top: mass spectrum; botton: list of peptide masses. (DOCX) [file pone.0111204.s005.docx]

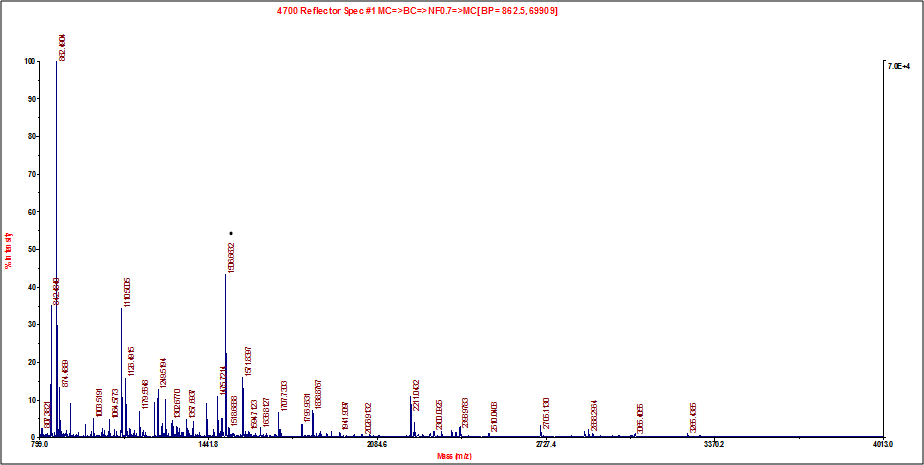


**MS masses list (m/z) and MS/MS (m/z) obtained experimentally:**

807.3821, 862.4904, 874.4889, 914.3996, 973.5053, 1003.5191, 1037.4944, 1064.5773, 1110.5005, 1126.4915, 1142.4908, 1179.5648, 1234.6422, 1248.6680, 1249.5194, 1263.6578, 1265.5255, 1307.6449, 1323.6357, 1329.6710, 1357.6937, 1379.6826, 1434.7362, 1460.7509, 1475.7214, 1491.7383, 1493.7217

1506.6632 ions(84.0706, 86.0837, 102.0224, 112.0633, 129.0797, 158.0543, 171.0703, 175.0791, 215.0742, 231.0675, 232.0849, 233.7614, 242.0553, 259.0272, 270.0061, 276.1004, 300.0969, 312.0817, 316.0601, 329.0672, 330.0758, 347.0696, 357.0575, 384.0276, 406.1142, 427.0736, 429.0905, 444.1158, 463.0685, 464.1406, 470.0504, 488.1243, 501.1241, 527.1346, 528.0889, 545.1414, 548.1027, 576.1713, 577.1082, 593.1036, 630.1601, 634.1406, 656.1445, 674.1600, 720.1392, 728.2151, 803.1410, 807.2059, 815.1727, 817.2496, 833.1819, 847.2329, 914.1097, 914.5057, 915.2222, 931.2125, 932.1835, 934.3376, 962.2170, 965.3189, 980.1932, 1019.2691, 1035.2124, 1045.2312, 1061.2220, 1063.2351, 1078.2394, 1161.3419, 1207.2797, 1209.4647, 1275.2233, 1332.2047, 1336.3109, 1350.1788, 1378.3833, 1393.2719, 1444.9957, 1445.8665, 1449.7865, 1461.8837)

1518.6888, 1571.8397, 1593.7123, 1638.8127, 1707.7303, 1716.8081, 1796.9631, 1838.8767, 1862.9840, 1867.8607, 1908.7566

**Supplementary material 5A.** *MvFABPa mass spectrometry identification of tetrathyridia cytosolic fraction.*

Top: mass spectrum; botton: list of peptide masses.


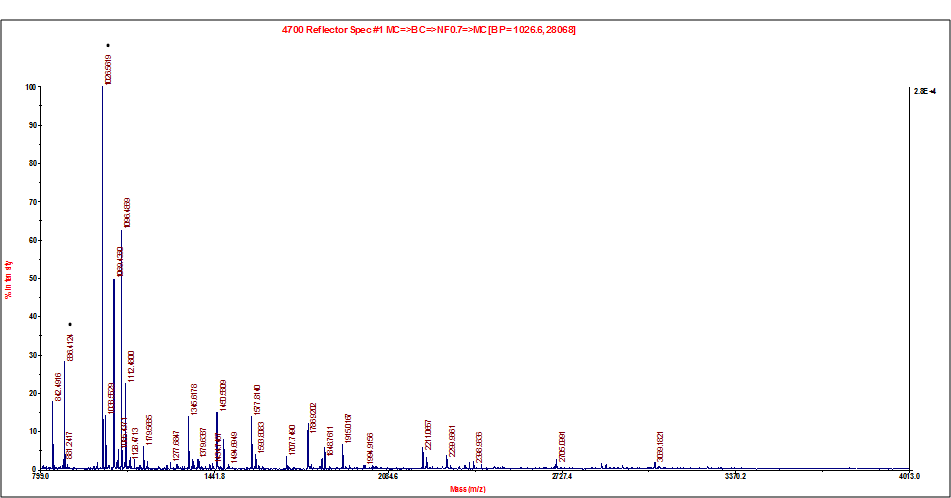


**MS masses list (m/z) and MS/MS (m/z) obtained experimentally:**

881.2417, 886.4124 ions(175.1008, 195.0869, 198.0750, 207.0155, 216.0857, 232.1281, 249.1018, 255.1072, 276.1519, 294.1633, 327.1154, 345.1161, 358.1744, 360.1204, 363.1239, 363.6226, 377.1684, 378.1288, 395.1963, 403.1296, 428.1622, 439.2510, 446.1521, 464.1561, 475.1930, 492.1710, 501.2471, 524.2232, 542.2468, 548.2029, 550.0456, 558.2000, 565.2194, 575.1786, 577.0596, 577.9098, 581.2516, 586.2072, 593.2042, 653.2932, 671.2637, 697.8549, 704.1841, 711.9418, 722.2634, 723.2485, 740.2486, 754.3118, 772.3192, 774.3101, 782.3116, 801.2307, 824.4494, 842.2367, 843.0779, 851.0608)

1026.5619 ions(70.0432, 86.0764, 110.0389, 112.0591, 129.0800, 138.0248, 157.0548, 158.0555, 175.0695, 223.0872, 229.0575, 234.0558, 246.0803, 251.0746, 261.0821, 262.0363, 270.1107, 271.0594, 290.0635, 308.0808, 342.1063, 357.1135, 359.1303, 361.0760, 375.0900, 379.1156, 382.0437, 384.0924, 390.0888, 407.1096, 418.0799, 424.1302, 486.1176, 489.1327, 490.1137, 503.1438, 504.0884, 506.1399, 521.1107, 531.1189, 586.1054, 603.1300, 620.1440, 621.1226, 630.1219, 640.1630, 642.9391, 651.1100, 668.1378, 716.1463, 719.1812, 735.1761, 742.1025, 753.2200, 755.9772, 759.1570, 776.1722, 781.2048, 789.0718, 824.1658, 826.9482, 852.1798, 854.0800, 854.9779, 869.1951, 869.3525, 870.1824, 889.2170, 967.1000, 984.0049, 990.9203, 1008.6237, 1008.9313)

1038.5529, 1069.4360, 1078.4913, 1081.4702, 1085.4371, 1096.4869, 1112.4800, 1128.4713, 1144.4908, 1179.5685, 1193.5905, 1277.6847, 1345.6178, 1361.6078, 1379.6387, 1383.6484, 1421.7150, 1434.7467, 1450.5809, 1475.7224, 1494.6949, 1577.8140, 1591.7681, 1593.8083, 1707.7490, 1786.9202, 1798.9556, 1838.8911, 1848.7611, 1915.0167

**Supplementary material 5B.** *MvFABPb mass spectrometry identification of tetrathyridia cytosolic fraction.*

Top: mass spectrum; botton: list of peptide masses.
